# Utilization of semiconductor-decorated tungsten trioxide nanowire membranes for the elimination of microorganisms from potable water

**DOI:** 10.1039/d5ra04826e

**Published:** 2025-08-12

**Authors:** Mohammed Ahmed Shehab, Emma Szőri-Dorogházi, Tímea B. Gerzsenyi, Munaf Al-lami, Andrea Valsesia, Tamás Szabó, Khalid T. Rashid, Adnan A. AbdulRazak, Anett K. Leskó, Haidar Hasan Mohammed, Zoltán Németh

**Affiliations:** a Polymers and Petrochemicals Engineering Department, Basrah University for Oil and Gas Basrah 61004 Iraq mohammed.ahmed@buog.edu.iq; b Advanced Materials and Intelligent Technologies Higher Education and Industrial Cooperation Centre, University of Miskolc Miskolc H-3515 Hungary; c Department of Chemical Engineering and Petroleum Refining, Basrah University for Oil and Gas Basrah 61004 Iraq; d European Commission, Joint Research Centre (JRC) Ispra Italy; e Institute of Ceramics and Polymer Engineering, University of Miskolc Miskolc H-3515 Hungary; f Membrane Technology Research Unit, Department of Chemical Engineering, University of Technology-Iraq Alsinaa Street 52 Baghdad 10066 Iraq; g Department of Business and Entrepreneurship Development, Institute of Lorantffy, University of Tokaj Eötvös u. 7 Sárospatak H-3950 Hungary; h Thermodynamics and Mathematical Physics Unit, Faculty of Engineering, University of Mons Mons 7000 Belgium

## Abstract

Microbial contamination in drinking water continues to be a significant global issue due to its direct effects on human health, particularly in areas with insufficient sanitation or deteriorating infrastructure. Conventional treatment systems frequently encounter challenges in fully eliminating pathogenic bacteria, underscoring the pressing necessity for innovative, energy-efficient filtration technology to ensure universal access to clean drinking water. In this regard, numerous reconsidered membrane technologies and filtration solutions have been developed and published recently. In this study, tungsten trioxide nanowire (WO_3_)-based hybrid membranes were prepared using a hydrothermal method followed by a simple impregnation process. The present study shows the removal efficiency of WO_3_ NW-based hybrid membranes in the filtration experiments of *Escherichia coli* (*E. coli*) bacteria and MS2 bacteriophages. The surface of the as-prepared WO_3_ NW was decorated with iron(iii) oxide (Fe_2_O_3_) and copper(ii) oxide (CuO) nanoparticles, respectively, and cellulose was used as a reinforcement matrix during membrane preparation. Produced hybrid membranes were characterized by micro computed tomography (μCT), Raman spectroscopy, Fourier transform infrared spectroscopy (FT-IR), dynamic light scattering (DLS) and inductively coupled optical emission spectrometry (ICP-OES) techniques. The results showed that the WO_3_–Fe_2_O_3_–cellulose membrane achieved a 90% removal efficiency for *E. coli*. In comparison, the WO_3_–CuO–cellulose membrane demonstrated a 2.5 log reduction value (LRV), corresponding to a 99.7% removal efficiency for MS2 bacteriophage. The application of Fe_2_O_3_ and CuO nanoparticles for the surface modification of WO_3_ NW resulted in notable distinctions when comparing the results of the *E. coli* and MS2 filtering experiments. The observed phenomena can be attributed to electrostatic surface interactions between the membranes and microorganisms, as confirmed by zeta potential measurements. With this study, the authors aim to show a facile, eco-friendly and cost-effective membrane solution for the removal of microorganisms from drinking water.

## Abstract 2

Microbial decontamination using WO_3_ NW-based hybrid membrane.

## Graphical abstract

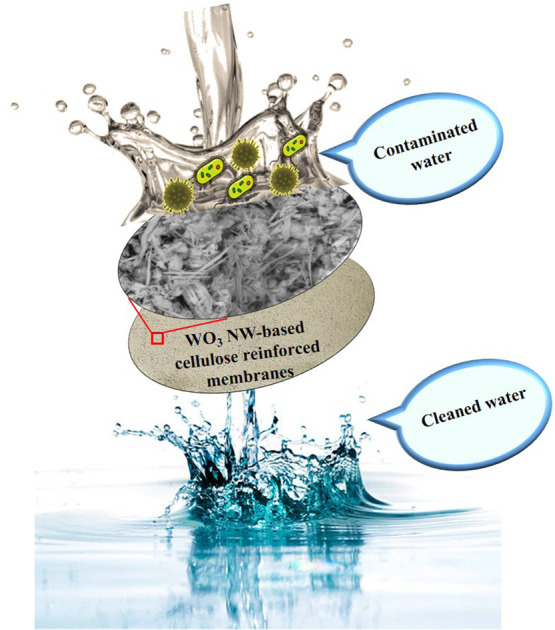

## 1. Introduction

Nowadays, wastewater treatment that includes the removal of traces of organic pesticides, dyes, and microorganisms (*i.e.*, bacteria, viruses, fungi) has received considerable attention worldwide. This is because it poses a major threat to all living organisms. The cases of contamination, especially with microorganisms are rising primarily due to changes in climatic conditions resulting in floods or other calamities in different parts of the world. This leads to contamination of our resources, including water. The presence of microorganisms, in particular Gram-positive and Gram-negative bacteria and viruses in an environment result in the spread of life-threatening diseases.^1,2^ Recent studies show that many efforts were accomplished to remove microorganisms from water using different techniques.^3–6^ Among these methods, membrane technology has been considered one of the most important methods to remove emerging contaminants including microorganisms due to unique features such as non-toxicity, easy to handle, and efficiency.^7^ Nevertheless, specific limitations, including pore blockage and fouling, can considerably decline membrane performance. The integration of functional nanomaterials into membrane structures has demonstrated efficacy in addressing these challenges and improving overall efficiency. Nanomaterials are highly recommended for water purification owing to their notable characteristics such as a large surface area, good mechanical properties, cost-effectiveness, lower energy demands, higher chemical reactivity, recyclability, ease of production, functionalization capabilities, as well as their ability to exhibit antiviral and antibacterial properties.^8,9^

Recently, tungsten trioxide (WO_3_) has gathered much attention of the material science chemist due to its response under visible light. It is a metal oxide semiconductor photocatalyst with a smaller band gap (∼2.8 eV) than TiO_2_ that makes it capable of absorbing larger portion of visible light spectrum. Its exceptional optical properties, large energy storage potential and stable physiochemical properties make it a diverse material with a range of applications including wastewater treatment, sensors and dye-sensitized solar cells.^10,11^ Furthermore, it has been widely studied and proved successful in biomedical applications. This is due to the large surface area, small size and high reactivity.

Pristine WO_3_ doesn't exhibit significant antimicrobial properties without light irradiation use. To validate this statement, G. Duan *et al.*, investigated the antibacterial activity of WO_3−*x*_ against both Gram-negative *Escherichia coli* and Gram-positive *Staphylococcus aureus* (*S. aureus*) strains with and without sunlight exposure. They found that almost 70% of enhancement in germicidal activity of pure WO_3−*x*_ was observed under sunlight exposure.^12^ With a view to further improving the germicidal response of pure WO_3_, several metal and metal oxide nanomaterials have been extensively studied due to their highly tunable structures and stoichiometry. To investigate the role of metal or metal oxide in improving the antibacterial properties, M. Matalkeh *et al.* synthesized Ag nanoparticles supported on WO_3_ and studied its antibacterial activity towards Gram-negative *E. coli* and the Gram-positive *S. aureus* under visible light and in dark.^13^ The result showed that Ag/WO_3_ exhibits strong antibacterial activity under visible light. In another study, M. Arshad *et al.* evaluated the antimicrobial activity of undoped WO_3_ and Zn-doped WO_3_ against different Gram-positive (*Bacillus subtilis* and *Staphylococcus aureus* and Gram-negative (*Escherichia coli* and *Pasturellamultocida*) bacterial strains.^14^ The results demonstrated that Zn-doped WO_3_ exhibited 20 to 25% higher antibacterial activity than pure WO_3_. Similarly, B. M. Alshabander studied the antibacterial activity of pure WO_3_ and Cu-doped WO_3_ against two bacterial pathogens, Gram-positive *Staphylococcus aureus* and Gram-negative *Escherichia coli* using light and dark mode.^15^ The results demonstrated that Cu-doped WO_3_ showed higher photocatalytic antibacterial activity than pristine WO_3_. In another work, U. Baig *et al.* prepared silver (Ag) decorated tungsten trioxide applying wet impregnation technique for inactivation of harmful water-borne Gram-negative pathogens. The study revealed that the proximity of nanomaterials to the bacterial cell wall and membrane caused significant membrane integrity disruption, ultimately leading to cell death.^16^ Although these metal nanomaterials have unique properties, but they have some drawbacks as well, such as, expensive, and, in some cases, can be toxic or unstable.^17^

For that, metal oxide nanoparticles have impressive features such as stability, cost-effectiveness, ease of availability and non-toxicity.^17^ Consequently, many researchers used metal oxides to improve antimicrobial activity of WO_3_. For instance, Y. L. Ying *et al.* synthesized WO_3_/ZnO and studied its antibacterial activity against Gram-positive bacteria (*Bacillus subtilis* and *Staphylococcus aureus*) and Gram-negative bacteria (*Escherichia coli* and *Pseudomonas aeruginosa*) The results showed that WO_3_/ZnO achieved a better inhibition effect against Gram-positive bacteria than Gram-negative bacteria.^18^ E. Falletta *et al.* prepared WO_3_/TiO_2_ composites with different ratios and investigated their *E. coli* inactivation performance under visible irradiation. The results demonstrated that WO_3_/TiO_2_ composites could not degrade *E. coli* under visible light irradiation.^19^ Another study investigated antimicrobial properties of WO_3_, graphene oxide (GO) and WO_3_/GO nanocomposite against (*E. coli*, *Pseudomonas aeruginosa* and *Candida albicans*) as reported by T. Muzaffar *et al.* They found that WO_3_/GO composite showed good antimicrobial activity compared to pure GO and WO_3_.^20^

These days, one of the main challenges in the fields of environmental protection and water purification is to develop hybrid membranes not only for selective removal of microorganisms but also to improve the reusable application possibilities, reducing their environmental footprint. In this regard, inorganic nanoparticles, such as iron(iii) oxide (Fe_2_O_3_) and copper(ii) oxide (CuO) have also received significant attention both in composite and membrane preparation, because they are cheap, safe, non-toxic, removal ability of organic pollutants, antibacterial and antiviral properties.^21,22^ They may have substantial potential to create heterojunction regions when combined with WO_3_. In order to prepare membrane, cellulose is often used as reinforcement material, due to its low cost, unique structure and biodegradability. As it was presented many times, cellulose has remarkable properties and is insoluble in most of the solvents, while the large number of hydroxyl groups on its surface makes it a valuable material in membrane technology applications.^23,24^ The membranes, which were built by the usage of these nanoparticles and different types of inorganic nanowires, such as TiO_2_ (ref. 25) or MnO_2_ (ref. 26) have already been investigated and published. However, to our best knowledge, the fabrication and utilization of WO_3_ NWs/CuO/cellulose and WO_3_ NWs/Fe_2_O_3_/cellulose membranes for the removal of microorganisms using gravity-driven filtration have not been reported. Furthermore, earlier research has examined WO_3_-based composites typically integrated with metals or metal oxides for antibacterial or antiviral properties, predominantly depending on light-assisted mechanisms. Nonetheless, their ability to operate as independent filtration membranes functioning under dark, inactive conditions has not been investigated.

This study aimed to evaluate the antibacterial and antiviral filtration abilities of the as-prepared hybrid membranes towards the *E. coli* and MS2 pathogens. In addition, a comparative analysis is presented between the filtration and removal efficiencies of WO_3_ nanowire (NW)- and TiO_2_ nanowire (NW)-based hybrid membranes.^25^ This study advances the field of water treatment and public health by introducing a cost-effective, gravity-driven filtration method utilizing innovative WO_3_ NWs-based hybrid membranes, which exhibit proven antibacterial and antiviral properties. Furthermore, microbial contamination in drinking water is a significant global issue, especially in low-resource environments where traditional disinfection techniques may be inaccessible or impractical. The creation of membranes possessing inherent antimicrobial characteristics that operate independently of external energy or chemical additives presents a sustainable, passive approach consistent with global initiatives aimed at enhancing water safety and mitigating the transmission of waterborne diseases.

## 2. Experimental section

### 2.1. Materials and reagents

Sodium tungstate dihydrate (Na_2_WO_4_·2H_2_O, 99%) was purchased from Thermo Fisher (Hungary), sodium hydroxide (NaOH, 99%) and ethanol (EtOH, 99.5%) were purchased from Sigma Aldrich (Hungary). The sodium sulfate (Na_2_SO_4_, 99%) was ordered from Lachner Ltd. (Czech Republic). Hydrochloric acid (HCl, 37%) and copper(ii) acetate monohydrate (Cu(OOCCH_3_)_2_·H_2_O, 98%) were ordered from VWR Chemicals (Hungary). Iron chloride hexahydrate (FeCl_3_·6H_2_O, 97%) was purchased from Scharlab (Hungary). Cellulose microfibers are originating from the DIPA Ltd (Hungary). For the preparation of hybrid membrane, polyvinylidene (PVDF) filter membrane with 0.1 μm pore size, 47 mm diameter and 125 μm thickness (Durapore-VVLP04700) used was ordered from Sigma Aldrich (Hungary).

The bacteriological agar, d-glucose and sodium dihydrogen phosphate dihydrate (NaH_2_PO_4_·2H_2_O) were obtained from Sigma-Aldrich (Switzerland). Glycerol, calcium chloride dihydrate (CaCl_2_·2H_2_O) and microbiology yeast extract were purchased from Merck Eurolab (Switzerland). Streptomycin was delivered by AppliChem PanReac (Germany). Sodium chloride (NaCl) and tryptone (Difco 0123) were bought from VWR International (Switzerland) and Becton Dickinson, respectively. The Gram-negative strain *Escherichia coli* DH5α (SZMC 21399) was used as a model organism for the filtration test (the bacterial strain utilized was donated by the Faculty of Science and Informatics, Department of Microbiology, at the University of Szeged). For MS2 bacteriophage multiplication (DSM No. 13767), *Escherichia coli* (Migula 1895) Castellani and Chalmers 1919 (DSM No. 5695) colonies were applied as host cells. The MS2 phage suspension and dry *E. coli* pellets were obtained from DSMZ (Braunschweig, Germany).

### 2.2. Synthesis of WO_3_ NW-based hybrid membranes

The preparation of WO_3_ NW, and the WO_3_ NW–Fe_2_O_3_ and WO_3_ NW–CuO composites were similar to the method presented in our previous study.^27^ In brief, hydrothermal method was applied for the production of WO_3_ NW, and a simple impregnation technique was then followed to decorate the surface of the as-prepared WO_3_ NW with Fe_2_O_3_ and CuO nanoparticles, resulting in the composites, namely, WO_3_ NW–Fe_2_O_3_ and WO_3_ NW–CuO, respectively.

Hybrid membrane synthesis was initiated by fabricating WO_3_ NW–Fe_2_O_3_ and WO_3_ NW–CuO composites. For this, in order to achieve a homogeneous suspension, the precursor compounds (0.0423 g of FeCl_3_·6H_2_O and 0.1255 g of Cu(CH_3_COO)_2_·H_2_O) were first dissolved in 100 mL of distilled water and 100 mL of EtOH, respectively. Afterwards, WO_3_ NWs were added to the precursor solutions, stirred for 1 h and placed in an autoclave. The resulting products were washed with distilled water using vacuum filtration to obtain a neutral pH and finally calcinated in a static oven at 500 °C for 2 hours. In all cases, the amount of Fe_2_O_3_ and CuO nanoparticles was 5 w/w% in the final composites.

To produce WO_3_ NW-based hybrid membranes, 0.2 g of the as-prepared composite was suspended in 100 mL of EtOH. The suspension was then homogenized for 1 h. Subsequently, a cellulose suspension containing 5 g of 1% (w/w) concentration was introduced into the mixture. The resulting hybrid membranes were fabricated using vacuum filtration, utilizing a PVDF membrane. Then, the membranes were subjected to drying for 30 min at 40 °C. The total weight of the membrane is 250 mg.^27^

## 3. Characterization

The YXLON FF35 dual-beam X-ray micro-computed tomography equipment (CT) with a microfocus X-ray tube, transmission beam, and an acceleration voltage of 50 kV was utilized to analyze the hybrid membranes' surface characteristics and 3D morphology.

The high-resolution Raman spectrometer used in this study to identify the chemical interaction between membrane components. It was the Nicolet Almega XR, manufactured by Thermo Electron Corporation in Waltham, MA, USA. Raman spectroscopy was employed as an analytical technique, with a 532 nm Nd:YAG laser operating at a power of 50 mW.

The electrophoretic measurement was conducted using a dynamic light scattering (DLS) instrument, namely the ZetaSizer NS-Malvern from the United Kingdom. The M3-PALS technology developed by Malvern utilizes a hybrid approach that combines laser Doppler velocimetry with phase analysis of light scattering (PALS). The measurement of the dissolved quantities of copper (Cu) and iron (Fe) during the filtering tests was conducted utilizing the inductively coupled optical emission spectrometry (ICP-OES) technique. This analysis was performed using a Varian 720 ES ICP-OES instrument.

The Fourier-transform infrared (FT-IR) spectra was obtained within the 4000 to 400 cm^−1^ range. The measurements were conducted with an optical resolution of 4 cm^−1^ using a Bio-Rad Digilab Division FTS65A/896 FT-IR spectrometer. The spectrometer had a deuterated triglycine sulfate (DTGS) detector and a germanium/potassium bromide (Ge/KBr) beamsplitter. The experimental setup involved the utilization of a Harrick's Meridian® SplitPea Single Reflection Diamond ATR attachment, which obviated the need for any sample preparation. The spectrometer was operated using Win IR Pro v. 3.3 software, developed by the Bio-Rad Digilab Division. The obtained spectra were analyzed using GRAMS/AI v. 7.0 software developed by Thermo Galactic. FTIR analysis was also conducted to confirm the presence of chemical interactions between the membrane components.

Considering the length of the present work, the other detailed study on the characterization part of the samples such as scanning electron microscopy (SEM), transmission electron microscopy (TEM), X-ray powder diffraction (XRD), and specific surface area measurements (N_2_-adsorption) are not presented here. However, our previous study on the WO_3_ NW based hybrid membranes for photocatalytic removal of methylene blue dye could be found here.^28^

### 3.1. Membrane porosity and pore size

The porosity and pore size were estimated using the following eqn (1) and (2), which are based mainly on the permeate flux and the properties of the prepared membranes: wet membranes (1 cm^2^) were initially weighed, and then they completely dried in a desiccator at 40 °C. The total porosity (*ε*) of each membrane was determined using eqn (1).1
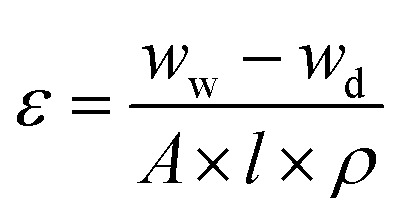


The average pore radius (*r*_m_) was estimated using the Guerout–Elford–Ferry equation (eqn (2)):2
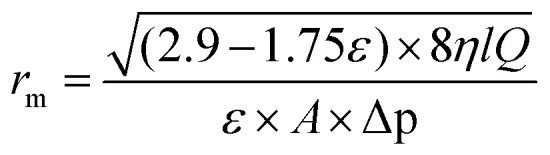
where *ε* is porosity, *w*_w_ and *w*_d_ are wet and dry membrane weight, *A* is the area of membrane, *l* is the thickness of membrane, *ρ* is pure water density, *Q* is the volume of collected pure water per unit time (m^3^ min^−1^), Δ*p* (1 bar) refers to the operation pressure, and *η* represents the water viscosity (8.9 × 10^−4^ Pa s). In order to minimize experimental errors, the final property values of each membrane were calculated by averaging the results of three samples.

## 4. Measurements of bacteria and virus filtration, and toxicology tests

### 4.1. Bacterial filtration

A series of tenfold dilutions were performed on the initial starter culture to evaluate the efficacy of bacterial filtration. This dilution was done in a 30 mL Lysogeny Broth (LB) medium. The concentration of colony-forming units per milliliter (CFU mL^−1^) in the sixth tenfold dilution was 1.21 ± 0.24 × 10^3^ CFU mL^−1^, determined using the colony counting method. Subsequently, the suspension mentioned above was employed for the filtration experiment, ensuring a discernible quantity of colonies on the agar plate. To determine the initial bacterial concentration in the suspension (CFU_0_), 3 × 50 mL of the sample was uniformly distributed onto LB agar plates prior to the filtration experiments. Filtration was then performed using a sterilized glass vacuum filtration apparatus (Sartorius Stedim Biotech 16306 – 25 mm), operated under gravity instead of vacuum. A total of 3 × 50 mL of the bacterial suspension was filtered at a rate of approximately 25 mL min^−1^. As a control experiment, we filtered the bacterial suspension just through the sterilized funnel (without any membrane) to assess the potential impact of the apparatus on bacterial removal efficacy. All membranes and equipment were sterilized before use with 70% ethanol, followed by dry heat treatment at 80 °C for one hour. Subsequent to filtering, the filtrates were put onto LB agar and incubated at 37 °C for 24 hours under aerobic conditions to estimate CFU counts. Following the filtration process, a volume of 3 × 50 mL was extracted from the filtrate to determine the quantity of colony-forming units (CFU_f_) present. The bacterial filtration efficiency (BFE%) was calculated using the following equation:
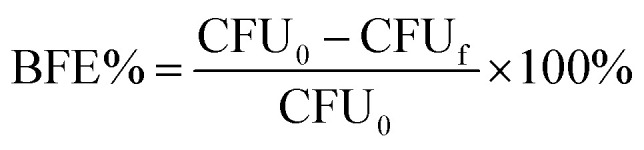


### 4.2. Virus filtration

The media (such as hard and soft agar, culture medium, antibiotic solution, virus dilution buffer) crucial for the culture and filtration of *E. coli* and MS2 were published by B. M. Pecson *et al.*^28^ The MS2 bacteriophage was cultured with the *Escherichia coli* host strain and then purified and concentrated according to the supplier's technique. Regular determination of plaque-forming units (PFUs) in the suspension was necessary due to the high sensitivity of MS2 bacteriophages. The initial concentration of the purified MS2 stock was determined to be 7.6 × 10^7^ plaque-forming units per milliliter (PFU mL^−1^). The substance was diluted using a viral dilution buffer (VDB) composed of NaH_2_PO_4_·2H_2_O, NaCl, and water to facilitate future applications. Hence, a sample amount of 2 mL was employed to determine a log reduction value (LRV) of approximately 4 or a detection limit of 99.99% for phage enumeration. The solution's pH was modified by adding a 0.1 M NaOH solution. The virus was stored at room temperature (23 °C) during the virus filtering studies. The concentration of MS2 bacteriophages was determined subsequent to the incubation period. The enumeration of MS2 samples was conducted twice, verifying each condition three times.

During the filtration investigations, the hybrid membranes that had been developed earlier were placed within a 200 mL glass funnel equipped with a porous glass insert at its base. As previously stated, gravity filtration was employed during the experimental procedures, thus preventing the need for a vacuum pump to enhance the flow rate of the filtration. To evaluate the effect of the device on bacteriophage retention, it was imperative to conduct a control experiment in the absence of a filter, considering the porous nature of the system. A volume of 30 mL of the viral dilution buffer was combined with the appropriate quantity of bacteriophage stock, resulting in an active phage concentration of 10 plaque-forming units (PFU) per milliliter. This methodology examined retention levels spanning 2 orders of magnitude (2 log). Clearly, distinct tests were carried out utilizing membranes composed of WO_3_ NW–Fe_2_O_3_ and WO_3_ NW–CuO composites. Following each filtration, 1 mL of the filtrate was collected and subsequently used to enumerate *E. coli* viruses. Filtration studies were conducted under conditions mimicking real-life scenarios, with a pH of 7.0.

### 4.3. Toxicological experiments

The industrial collaborator, Kisanalitika Ltd, located in Sajóbábony, Hungary, conducted the toxicological analysis. Fish tests have demonstrated efficacy in assessing the environmental risk associated with surface waters and determining the toxicological impacts of pollution. Acute toxicity refers to the manifestation of detrimental effects in an organism shortly after exposure to a substance, often occurring within days. Acute toxicity is commonly quantified as the lethal 50 (LC50) concentration. It refers to the quantity of the poisonous agent present in water that results in mortality in 50% of the tested group of fish within a designated timeframe. We performed an acute toxicity assessment by exposing zebrafish (*Danio rerio*) to membrane samples and observing survival and behavioral responses over short periods of time. No fatalities were noted, indicating that the membranes do not demonstrate acute toxicity under the conditions examined. In the experiments, a static mode of operation was employed, wherein the test solution remained unchanged, and was not renewed throughout the testing. A crucial factor to consider is the requirement for the fish to exhibit optimal health and the absence of any observable physical abnormalities. Water toxicity experiments were conducted by employing a dilution series, wherein water from the aquarium was utilized for dilution. This same water also served as the medium for the control test group. The diluting water within the fish tank underwent constant aeration, and regular monitoring was conducted to assess its oxygen concentration and pH level. The experimental groups were immersed in a 500 mL volume of filtrate water, which had been previously soaked with the hybrid membranes for 24 hours. Each experimental group consisted of five fish. As mentioned earlier, the fish specimens were immersed in a controlled water bath for 48 hours, with varying concentration levels. This study recorded fish mortality at regular intervals of 2 hours, 24 hours, and 48 hours.

## 5. Results and discussion

### 5.1. Micro CT analysis

**Fig. 1:**
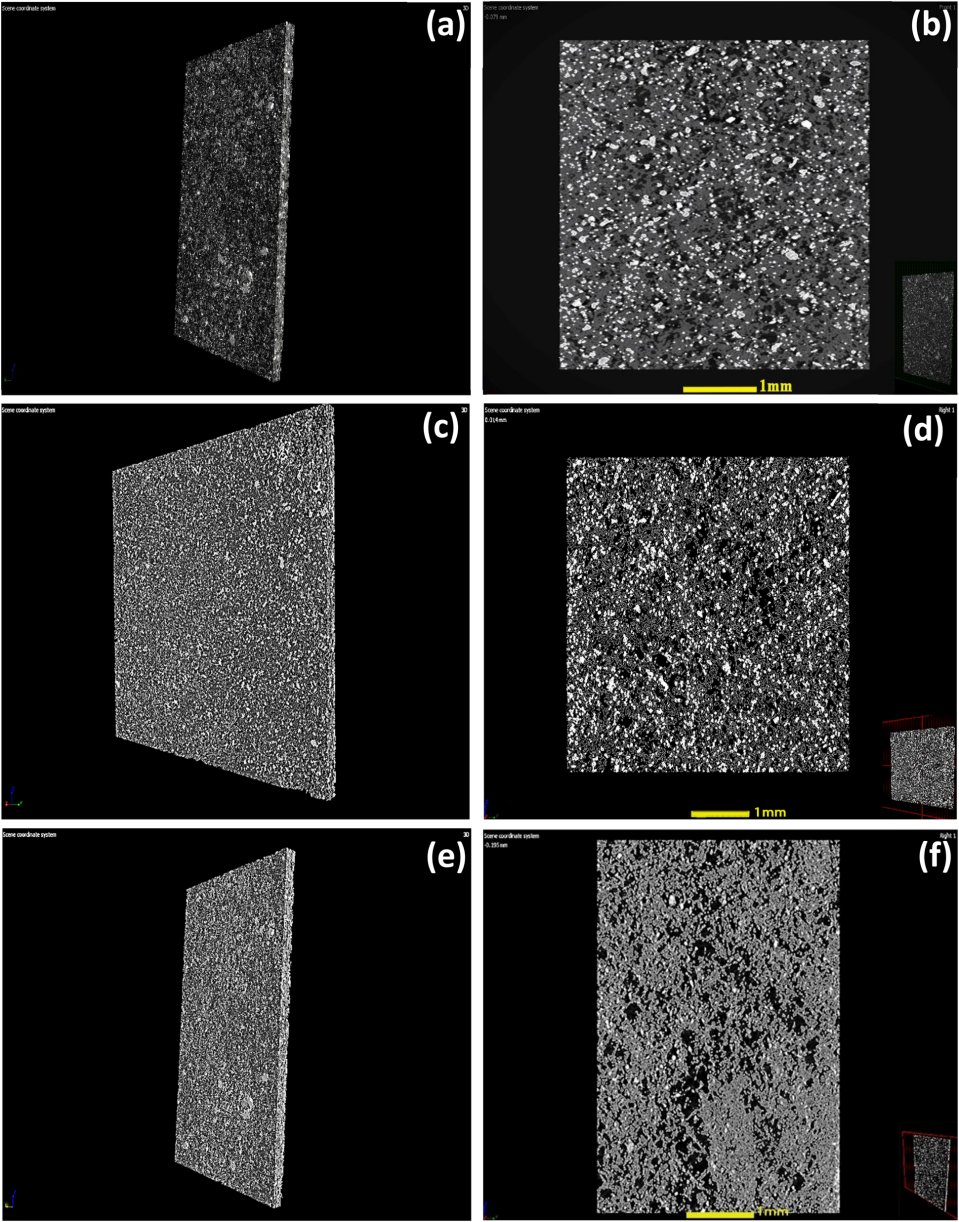
Three-dimensional computed tomography (CT) cross-section analysis of hybrid membranes: WO_3_ NWs–cellulose (a and b), WO_3_ NW–Fe_2_O_3_–cellulose (c and d) and WO_3_ NW–CuO–cellulose (e and f).

In our recent published study, we have provided analysis of transmission electron microscopy (TEM) and scanning electron microscopy (SEM) findings related to composites based on WO_3_ nanowires (NWs) and hybrid membranes.^29^ During the electron microscopy analyses, we gained information about the nanostructure of the composites and the surface nature of cellulose reinforced membranes. The confirmation on the formation of nanowires with average length of 0.8–3 μm were obtained from HRTEM and SEM. However, these techniques were not enough to provide insight from the 3D microstructure of the WO_3_ NW-based hybrid membranes. The electron microscopy techniques have a limitation which is the investigated membrane area comprises of a very small portion of the whole membrane that does not allow for us to get an extended overview about the homogeneity of membranes. Therefore, to overcome these limitations and to present 3D morphology of the membranes, micro-CT analysis was conducted. Fig. 1 depicts the 3-dimensional cross-sectional examination of WO_3_ NW–cellulose (Fig. 1a and b) WO_3_ NW–Fe_2_O_3_–cellulose (Fig. 1c and d) and WO_3_ NW–CuO–cellulose membranes (Fig. 1e and f).

In these subfigures, cellulose is represented as the brighter form, while the grey fibrous material is observed to originate from the WO_3_ NWs, WO_3_ NW–Fe_2_O_3_ and WO_3_ NW–CuO components. Fig. 1 illustrates the formation of a homogeneous distribution of composites and cellulose in the hybrid membrane structure. In order to further justify the homogeneity among the hybrid membranes, the volume of materials was measured. The values were ascertained through the full three-dimensional extension of the membranes and then documented in Table 1. The WO_3_ NW–cellulose membrane exhibited a material volume percentage of 43.8%, whereas the WO_3_ NWs–Fe_2_O_3_–cellulose membrane had a percentage of 47.5%. Meanwhile, the observed value for the WO_3_ NWs–CuO–cellulose membrane was 36.7%. According to the results above, it can be inferred that the 3D configuration of the as-prepared membranes appears the same. However, electron microscopy reveals noticeable differences in morphology, precisely material volume ratios. These differences may contribute to the difference in the efficiency of the membranes during the bacteria and virus filtration experiments. This will be examined in further sections.

**Table 1:** Values and characteristics of prepared membranes

Membrane	Material volume (mm^3^)	Material volume percentage (%)	Membrane pore size (nm)	Membrane porosity (%)
WO_3_ NW–cellulose	2.68	43.8	82.45	41.36
WO_3_ NW–Fe_2_O_3_–cellulose	2.42	47.5	79.5	55.48
WO_3_ NW–CuO–cellulose	1.12	36.7	84.27	53.26

### 5.2. Membrane porosity

**Fig. 2:**
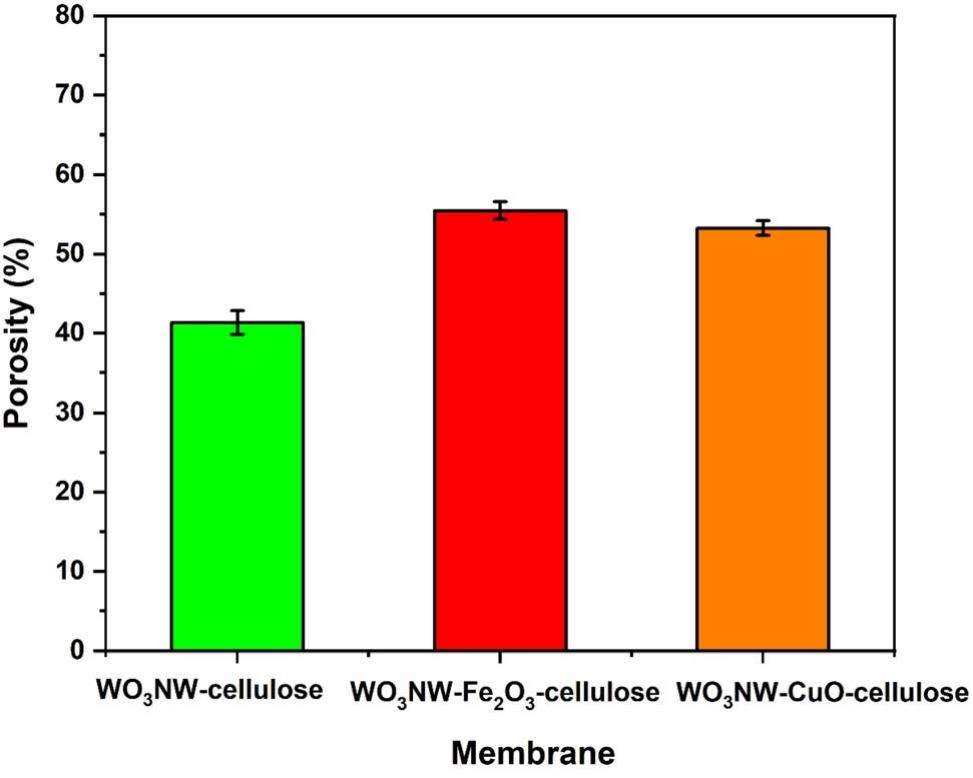
Effect of Fe_2_O_3_ and CuO on the membrane porosity.

Three distinct WO_3_ NW-based cellulose composite membranes are shown in Fig. 2 with their porosity (%): WO_3_ NW–cellulose (green column), WO_3_ NW–Fe_2_O_3_–cellulose (red column), and the WO_3_ NW–CuO–cellulose (orange column). As can be seen the WO_3_ NW–cellulose has the lowest porosity with 42%. On the contrary the WO_3_ NW–Fe_2_O_3_–cellulose has the highest porosity with 56%. Although the porosity of WO_3_ NW–CuO–cellulose has somewhat lower (∼54%) than that of the Fe_2_O_3_ variety, it is still much greater than that of WO_3_ NW–cellulose alone. The standard deviations in Fig. 2 indicate consistent and trustworthy measurements across replicates. Fe_2_O_3_ or CuO added to WO_3_ NW–cellulose enhances the porosity of the membrane, potentially improving permeability, diffusion rate, or surface area – properties that are crucial for catalysis, filtration, or sensing. In this combination, Fe_2_O_3_ seems to have a greater impact on porosity growth than CuO.

### 5.3. Raman analysis

**Fig. 3:**
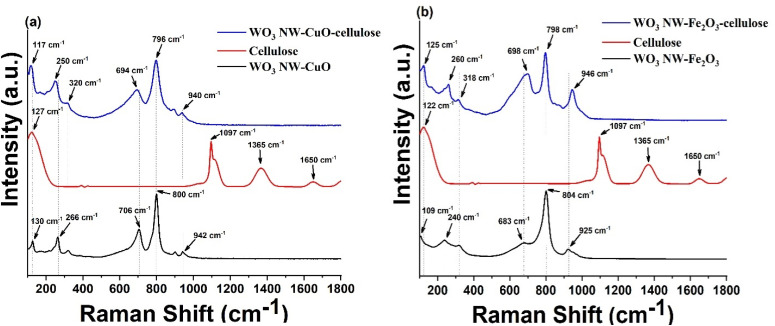
Raman spectra of (a) WO_3_ NW–CuO–cellulose, (b) WO_3_ NW–Fe_2_O_3_–cellulose hybrid membranes.

The presence of a chemical interaction between the cellulose reinforcement material and WO_3_ NW-based composites was postulated. To address this assumption, an analysis using Raman spectroscopy was conducted. Fig. 3a and b presents the Raman spectra of WO_3_ NW–CuO and WO_3_ NW–Fe_2_O_3_ composites, as well as the pristine cellulose and the WO_3_ NW–CuO–cellulose and WO_3_ NW–Fe_2_O_3_–cellulose hybrid membranes. The black curves represent the composites, the red curves represent the pristine cellulose, and the blue curves represent the hybrid membranes.

The Raman spectra depicted in Fig. 3a exhibit distinct peaks at 130, 320, and 706 cm^−1^, which can be attributed to specific vibrational modes. The peak at 130 cm^−1^ corresponds to the formation vibration of O–O bonds, while the peak at 320 cm^−1^ is associated with the bending vibration of bridging oxygen in W–O–W bonds. Additionally, the peak at 706 cm^−1^ represents the stretching vibration of bonds.^29^ The most prominent peak observed at 800 cm^−1^ can be attributed to the stretching vibration of O–W–O bonds. The peak observed at 940 cm^−1^ can also be attributed to the presence of –W=O bonds in hexagonal WO_3_ nanowires.^30^ The peaks at 266 cm^−1^ belonging to the CuO nanocrystals.^31^ The distinctive peaks corresponding to cellulose are observed at 127, 1097, 1365, and 1650 cm^−1^, as shown in Fig. 2a (red curve).^32^ In Fig. 3a, it can be observed that most peaks in the spectrum of the hybrid membrane (blue curve) exhibited shifts in both the typical peaks of WO_3_ NW and the CuO nanoparticles.

The Raman spectra depicted in Fig. 3b of the WO_3_–Fe_2_O_3_–cellulose hybrid membrane exhibits distinct peaks at 109, 318, and 683 cm^−1^, which can be attributed to specific vibrational modes. Specifically, the peak at 109 cm^−1^ corresponds to the formation vibration of O–O bonds, while the peak at 318 cm^−1^ corresponds to the bending vibration of bridging oxygen in W–O–W bonds. Lastly, the peak at 683 cm^−1^ corresponds to the stretching vibration of bonds. The most noticeable peak observed at 804 cm^−1^ can be attributed to the stretching vibration of O–W–O bonds, whereas the peak observed at 925 cm^−1^ can be attributed to the presence of –W=O bonds in hexagonal WO_3_ nanowires. The 240 cm^−1^ peak corresponds to the *E*_g_ mode of α-Fe_2_O_3_ crystals.^33^ Comparably, the spectrum of the WO_3_–Fe_2_O_3_–cellulose hybrid membrane (Fig. 3b (blue curve)) exhibited small shifts in the characteristic peaks of the WO_3_–Fe_2_O_3_ composite and cellulose, similar to those observed in the WO_3_–CuO–cellulose membrane (Fig. 3a). The phenomena mentioned above can be elucidated by non-covalent interactions, specifically hydrogen bonding, between WO_3_–CuO, WO_3_–Fe_2_O_3_ composites, and cellulose fibres, leading to the observed shifts in band peaks.^5^

### 5.4. FT-IR spectroscopy

**Fig. 4:**
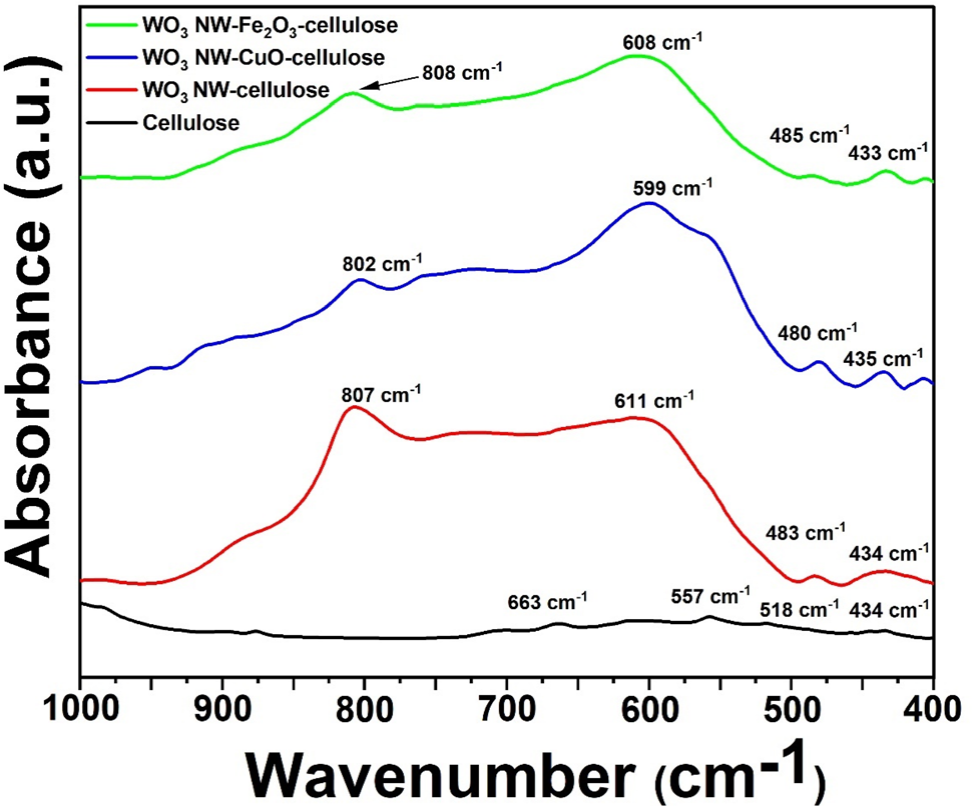
FT-IR spectra of cellulose (black curve), WO_3_–cellulose (red curve), WO_3_ NW–CuO–cellulose (blue curve) and WO_3_ NW–Fe_2_O_3_–cellulose (green curve).

To further investigate the aforementioned chemical interaction between the components of the membrane and the formation of chemical bonds, Fourier Transform Infrared (FT-IR) analysis was conducted. In Fig. 4, the infrared spectra of the hybrid membranes (blue and green curves) are presented within the 400 to 1000 cm^−1^ range, alongside the reference cellulose (black curve) and WO_3_ NW/cellulose (red curve). The lower frequency range mainly encompasses the vibrational bands associated with metal–oxygen stretching. The black curve indicates the cellulose sample exhibits four distinct bands at 434, 518, 557, and 663 cm^−1^. In contrast, the WO_3_ NW/cellulose composite (represented by the red curve) displays four bands with significant intensity at 434, 483, 611, and 807 cm^−1^. In the context of hybrid membranes, it is observed that the previously mentioned peaks exhibit slight shifts. Specifically, the peak at 434 cm^−1^ shifts to 435 and 433 cm^−1^, the peak at 483 cm^−1^ shifts to 480 and 485 cm^−1^, the peak at 611 cm^−1^ shifts to 599 and 608 cm^−1^, and the peak at 807 cm^−1^ shifts to 802 and 808 cm^−1^. These shifts are likely attributed to the chemical interaction between the WO_3_ NW/CuO, WO_3_ NW/Fe_2_O_3_ composites, and cellulose fibers.

### 5.5. Zeta potential

The zeta potential of the prepared samples was done to see the correlation between the surface charge and efficiency of the membranes. Table 2 displays the results of zeta potential (*ζ*) assessments conducted at a pH level of 7.0. The samples were initially dispersed in deionized water in order to attain a final concentration of 0.1% by weight before conducting the experiments. All measurements were repeated three times.

**Table 2:** Presents the values of zeta potential for cellulose and WO_3_ NWs-based membranes at a pH of 7.0

Sample	Zeta (*ζ*) potential (mV)
Cellulose	−32.2 ± 1.1
WO_3_ NWs–cellulose	−21.4 ± 0.8
WO_3_ NWs–Fe_2_O_3_–cellulose	−14.5 ± 0.5
WO_3_ NWs–CuO–cellulose	−7.6 ± 0.3

Comparative analysis of *ζ*-potentials of cellulose, WO_3_ NWs–cellulose and hybrid membranes demonstrated that the virus retention efficiency can be enhanced *via* CuO and Fe_2_O_3_ nanoparticles attached to the surface of WO_3_ NWs. A negative zeta potential value was found for pure cellulose (−32.2 mV) while the hybrid membranes are proved to be more positively charged (−14.5 and −7.6 mV) in consequence of functionalization. The surface treatment of the hybrid membranes leads to a shift in the isoelectric point (IEP) towards higher values, resulting in an increase in positive charge on the surface. Consequently, the enhanced electrostatic interaction between the hybrid membrane surface and the bacteriophages may improve the filtration efficiency of MS2. The *ζ* potential of MS2 is estimated to be roughly −30 mV,^34^ a pure cellulose with a *ζ*-potential of −32.2 mV most probably will not be effective in a virus retention experiment.

On the contrary, Fe_2_O_3_ and CuO nanoparticles showed positive *ζ*-potential at pH 7.0, as reported by Kosmulski.^35^ Consistent with this, the findings presented in Table 2 demonstrate that incorporating CuO and Fe_2_O_3_ nanoparticles for surface functionalization of WO_3_ NWs resulted in a change towards fewer negative zeta-potential values in the hybrid membranes, as expected. These results indicate that the surface electrical properties of the WO_3_ NW-based membranes made them more advantageous for MS2 bacteriophage retention.

Electrostatic interactions, which are controlled by the membrane's and the virus particles' surface charge characteristics and *ζ*-potential (zeta potential), have a major impact on the retention of the microorganism when utilizing membranes based on tungsten trioxide nanowire (WO_3_ NWs). This is a thorough explanation of the relationship between these attributes: the electric potential at a particle or membrane surface's sliding plane in a fluid is represented by the *ζ*-potential. It serves as a stand-in for stability and surface charge in colloidal systems. Strong electrostatic repulsion or attraction with oppositely charged species is indicated by a high negative or positive *ζ*-potential. At physiological pH (∼7.4), viruses and bacteriophages often have a net negative charge because of their envelope proteins and glycoprotein spikes. Its *ζ*-potential normally varies between −10 and −25 mV, contingent on the presence of proteins or salts, pH, and ionic strength. According to the data in Table 2, WO_3_ NWs are negatively charged and electrostatically repel viruses, which might reduce adsorption until van der Waals forces or physical trapping take over.

### 5.6. Contact angle

Contact angle is one of the important parameters that impact membrane filtration efficiency. For that, contact angle measurements were utilized to determine the hydrophilic and/or hydrophobic properties of the as-prepared membranes. The application of water droplets on the membrane surfaces resulted in significant absorption, indicating that the membranes have a superhydrophilic nature. The distribution and alignment of CuO nanoparticles within the WO_3_ nanowire–cellulose matrix can affect the overall surface energy, which influences the contact angle. CuO nanoparticles can make the membrane's surface rougher. The hydrophilic surface's wettability is improved by increased roughness, which in turn increases hydrophilicity. CuO is inherently hydrophilic due to its surface energy and ability to form hydrogen bonds with water molecules. When CuO is added to the membrane, it introduces more active hydrophilic sites, such as surface –OH groups. Because cellulose has a large number of hydroxyl groups, it is inherently hydrophilic, whereas WO_3_ is somewhat hydrophilic. Presumably, CuO can work in concert with both, resulting in a more even distribution and exposure of hydrophilic domains, which could further increase water affinity even more.^36–38^

As for the inclusion of iron oxide (Fe_2_O_3_) in the cellulose nanowire membrane; Fe_2_O_3_ often affects hydrophilicity *via* altering surface chemistry, roughness, and electrical interactions. Due to its surface hydroxyl groups, which may interact with water molecules, Fe_2_O_3_ is naturally hydrophilic, particularly when it is in nanoparticle form. By increasing the density of these hydroxyl groups on the membrane's surface, Fe_2_O_3_ improves water adsorption and reduces the contact angle, a crucial indicator of hydrophilicity. It is clear from the above, the membrane's surface roughness may be increased at the nanoscale by adding Fe_2_O_3_ nanoparticles. The Wenzel model states that if the surface is already hydrophilic, adding roughness to it further decreases the water contact angle, increasing hydrophilicity.

### 5.7. Membrane permeate flux

**Fig. 5:**
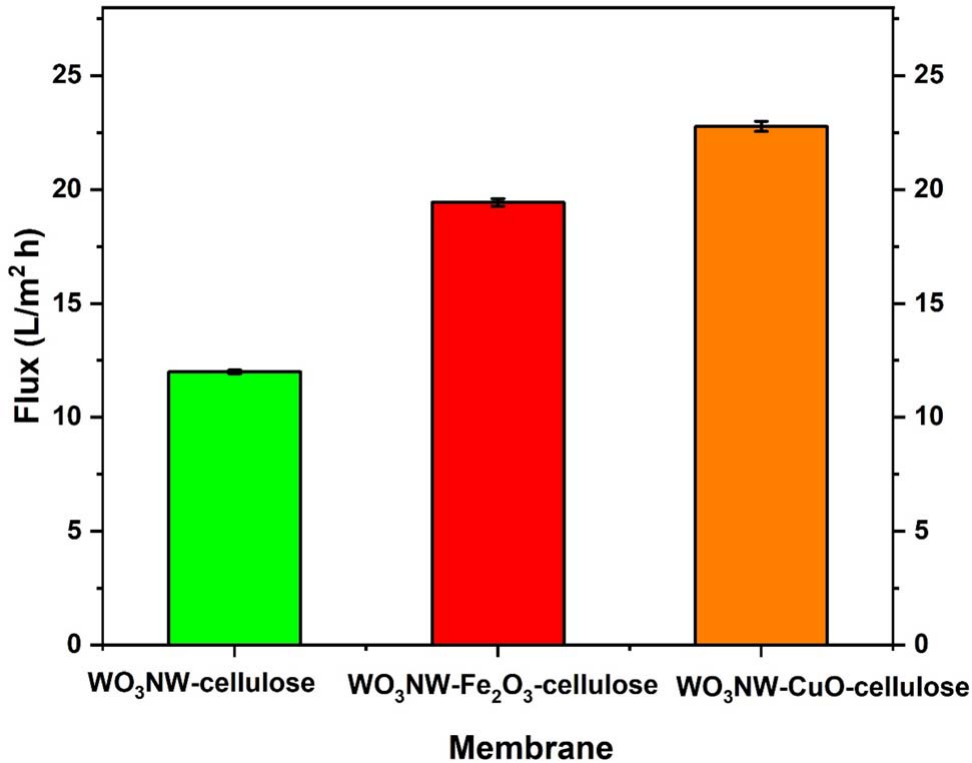
Effect of Fe_2_O_3_ and CuO NPs on the membrane permeate flux.

The effect of Fe_2_O_3_ and CuO nanoparticles (NPs) on the membrane permeate flow (measured in L m^−2^ h^−1^) for various WO_3_ NW–cellulose composite membranes is shown in Fig. 5. The lowest flux, around 12 L m^−2^·h^−1^, is seen in WO_3_ NW–cellulose membranes without additional metal oxide nanoparticles. When Fe_2_O_3_ is added to a WO_3_ NW–Fe_2_O_3_–cellulose membrane, the flow rises to around 19 L m^−2^·h^−1^. The WO_3_ NW–CuO–cellulose membrane has the greatest flux, at around 23–24 L m^−2^·h^−1^. The permeate flux of the membrane is greatly increased by the addition of Fe_2_O_3_ and CuO NPs. This enhancement is probably the result of the integration of nanoparticles, which might decrease resistance to water flow by increasing hydrophilicity, surface roughness, or porosity. Compared to Fe_2_O_3_, CuO NPs provide a higher enhancement, indicating either better interaction with the cellulose matrix or more advantageous changes to the membrane's characteristics (such as bigger pores or improved water affinity).

### 5.8. ICP-OES investigation

For the determination of total dissolved Cu and Fe content, the inductively coupled optical emission spectrometry (ICP-OES) method was used. Samples for the measurement were prepared by filtration of deionized water through as-prepared membrane at pH 7.0 under the experimental circumstances detailed previously. The results shown in Table 3 revealed that the amount of dissolved metal ions in all cases, less than the WHO lower limit^39^ The concentration of copper in the water sample is below the recommended limit of 2 mg L^−1^. However, no health-based guideline value has been established for iron. As a result, copper (Cu) and iron (Fe) leaching during the filtration procedure remains within the limits set by international regulatory standards. Furthermore, the data obtained indicate that the leaching process does not significantly impact on the results of the filtration tests involving *E. coli* and MS2.

**Table 3:** Shows the dissolved amounts of Cu and Fe determined by the ICP-OES technique

Membrane	Cu (μg L^−1^)	Fe (μg L^−1^)
WO_3_ NWs–Fe_2_O_3_–cellulose	—	62 ± 00 010
WO_3_ NWs–CuO–cellulose	108 ± 00 003	—

## 6. Applications

### 6.1. Analysis of *E. coli* filtration

**Fig. 6:**
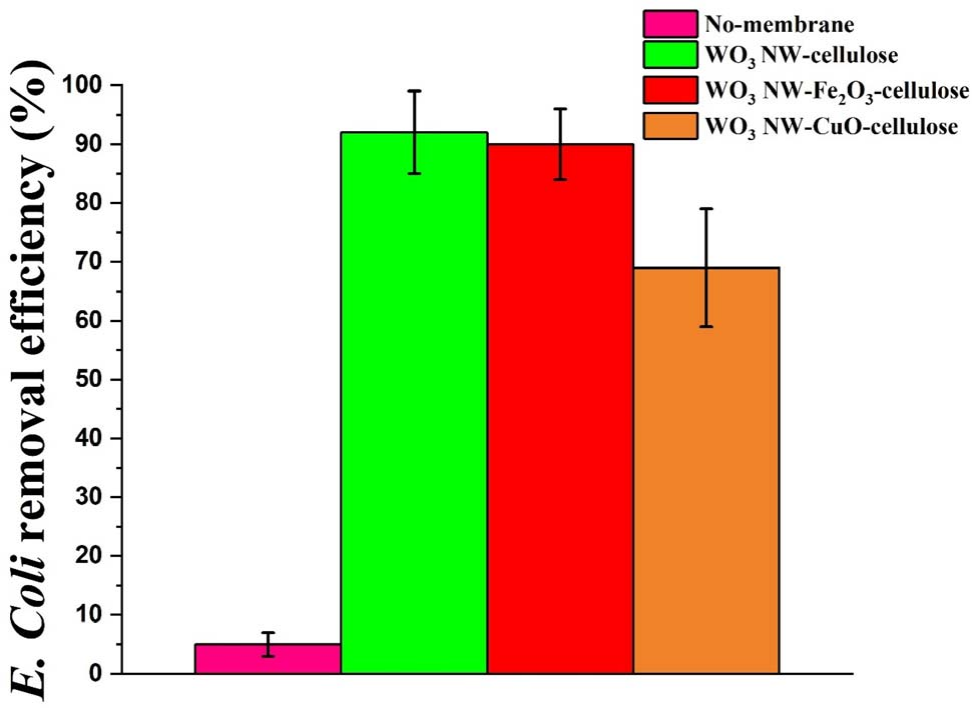
Bacterial filtration efficiency (BFE%) of WO_3_ NW-based hybrid membranes.

As already mentioned in the experimental section, before conducting the filtering tests, control measurements were performed in the absence of the as-prepared hybrid membranes in order to assess the potential risk of adsorption on the membrane holder of the funnel (as shown by the pink column in Fig. 6). The removal efficiency values of *E. coli* are depicted in Fig. 6. The results indicate that the membrane containing WO_3_ NW–Fe_2_O_3_ composite exhibited notably greater efficacy in removing *E. coli* (as shown by the red column in Fig. 6) compared to the membrane containing WO_3_ NW–CuO additive (as defined by the orange column in Fig. 6). On the other hand, the removal efficiency of WO_3_ NW–cellulose membrane (as shown by the green column in Fig. 6) (92%) was not significantly higher than WO_3_ NW–Fe_2_O_3_ membrane (89%). In contrast, the WO_3_–CuO–cellulose membrane showed lower *E. coli* retention with an effectiveness of 69%. These results showed a good correlation with the material volume percentages measured by 3D micro-CT technique and the porosity, as discussed earlier. Based on our previous studies,^25,40^ the *E. coli* filtration and removal process can be connected mostly with the size exclusion effect not to the electrostatic-based adsorption force. Consequently, membranes with higher material volume ratios, such as WO_3_ NW–cellulose membrane (43.8%) and WO_3_ NW–Fe_2_O_3_–cellulose (47.5%), are expected to provide higher *E. coli* retention values. Furthermore, the low porosity of the WO_3_ NW–cellulose membrane may serve as a physical barrier, effectively trapping *E. coli* and inhibiting its passage through the membrane.

Recently, we also presented the development and application of TiO_2_ NW-based hybrid membrane structures. During the *E. coli* experiments, the TiO_2_ NW–Fe_2_O_3_–cellulose membrane showed the highest retention values with an efficiency of 61%^25^ while the TiO_2_ NW–CuO–cellulose membrane provided lower value with 51%. Comparing the results of WO_3_-based and TiO_2_-based hybrid membranes,^25^ in general, it can be concluded that applying WO_3_-based membrane structures higher *E. coli* retention values can be reached in each case.

### 6.2. Analysis of MS2 filtration

**Fig. 7:**
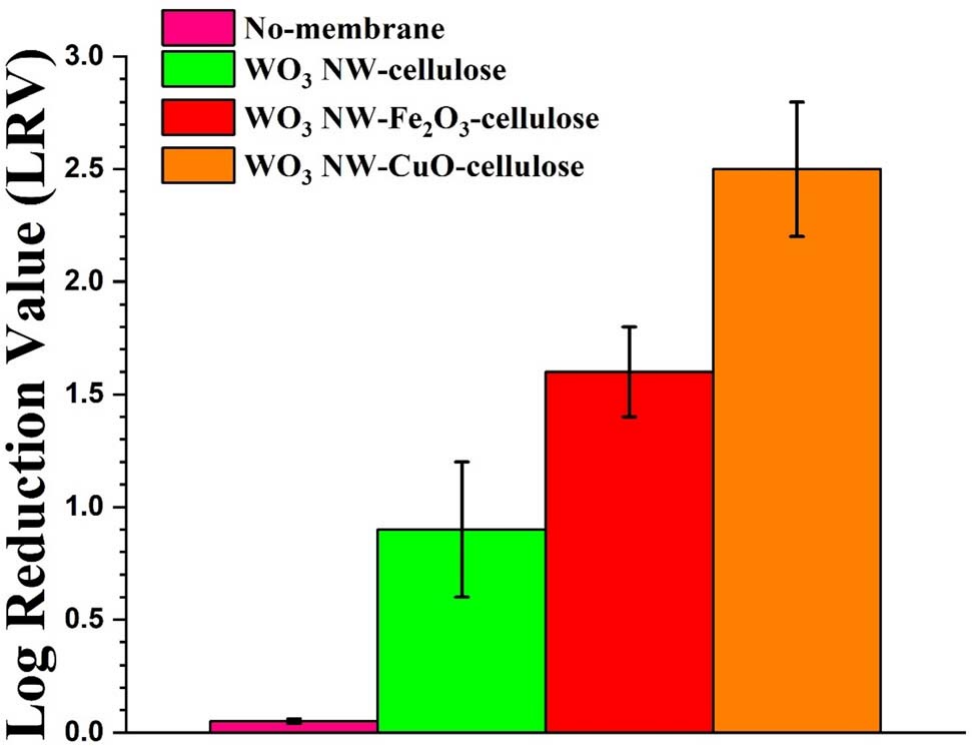
MS2 retention values of WO_3_ NW-based membranes in gravity driven filtration.

Fig. 7 illustrates the results of the MS2 filtering studies. These results confirmed that both membranes can retain MS2 bacteriophages used as pollutants models in this study; however, it was observed that the efficiencies of the two membranes differed significantly, as shown in Fig. 7. CuO-decorated WO_3_ NW-based membrane (orange column) had the most significant adsorption properties. When considering the pathogenic contamination, the water treatment efficiency is measured using log removal value (LRV). The WO_3_–CuO–cellulose membrane showed up to 2.5 LRV (log removal value) at pH 7 (orange column in Fig. 7) and the LRV values of both WO_3_–Fe_2_O_3_–cellulose and WO_3_–cellulose membranes showed considerably lower virus retention. Using WO_3_–Fe_2_O_3_–cellulose membrane (red column in Fig. 7), the virus retention value was observed at 1.6 LRV, whereas using a WO_3_–cellulose membrane without additional modification with nanoparticles resulted in a lower virus retention value of 0.9 LRV (green column in Fig. 7). On the other hand, the control experiment without a membrane (pink column in Fig. 7) did not exhibit any virus retention.

The importance of the substrate's specific surface area and the electrostatic force between bacteriophages and adsorbents has been previously published with effective virus rejection in virus filtering.^41^ The enhanced virus retention efficacy observed when using a WO_3_–CuO–cellulose membrane can be attributed to two primary mechanisms: the antiviral properties of CuO nanoparticles and their efficient surface adsorption facilitated by electrostatic forces.^42^ As discussed previously, the adsorption properties of TiO_2_ NW-based membranes against MS2 were also investigated. The study revealed high virus reduction value (up to 2 LRV) for the membrane containing CuO nanoparticles. Comparing the MS2 adsorption results of WO_3_-based and TiO_2_-based hybrid membranes, it was found that in each case the WO_3_-based hybrid membranes provided higher retention capacity. According to the data collected, it can be inferred that, in addition to the electrostatic interaction between the bacteriophages and the nano adsorbents, the material quality of the nanowire can significantly contribute in virus filtration.

### 6.3. Toxicology investigations

Toxicology testing is a crucial determinant influencing the usability and applicability of as-prepared membranes. The test revealed that none of the membranes demonstrated toxic properties under the specified experimental conditions (mentioned earlier) as shown in Table 4. Throughout the testing period, all fish groups demonstrated no mortality, indicating that the prepared membranes are non-toxic and environmentally friendly, thus representing a potential high-performance filter for water purification.

**Table 4:** Toxicological results of WO_3_ NWs-based hybrid membranes

Membrane	Death rate after 2 h	Death rate after 24 h	Death rate after 48 h
WO_3_ NWs/cellulose	0	0	0
WO_3_ NWs–Fe_2_O_3_/cellulose	0	0	0
WO_3_ NWs–CuO/cellulose	0	0	0

## 7. Conclusion

This study effectively proved the efficacy of WO_3_ nanowire (NW)-based hybrid membranes in eliminating microorganisms from water by simple gravity-driven filtering. The Fe_2_O_3_-coated WO_3_ NW membranes exhibited a bacterial removal efficiency over 90% against *E. coli*, whereas the CuO-coated WO_3_ NW membranes shown enhanced antiviral efficacy, achieving a log reduction value (LRV) of up to 2.5 against MS2 bacteriophage.

Toxicological assessments confirmed that WO_3_-based hybrid membranes do not exhibit any harmful toxic release, supporting their environmental safety and suitability for practical water treatment applications.

Compared to our previous research on TiO_2_ NW-based membranes, the WO_3_ NW-based membranes shown higher effectiveness in eliminating both bacteria and viruses. This enhancement results from the synergistic interaction between the metal oxides and the nanowires, associated with the enhanced positive surface charge that improves electrostatic interactions with negatively charged microbial membranes.

The overall performance demonstrated by the WO_3_ NW-based hybrid membranes suggests potential for future development of effective membrane materials for purifying water contaminated with microorganisms. Future research will focus on evaluating the long-term stability, reusability, and scalability of these membranes in practical applications.

## Author contributions

Mohammed Ahmed Shehab: conceptualization, methodology, validation, investigation, writing – original draft, writing – review & editing. Emma Szőri-Dorogházi: methodology, validation, investigation, writing – review & editing. Tímea B. Gerzsenyi: investigation, visualization. Munaf Al-lami: formal analysis, resources. Andrea Valsesia: investigation, visualization. Tamás Szabó: investigation. Khalid T. Rashid: investigation, validation, formal analysis, writing – review & editing. Adnan A. AbdulRazak: investigation, formal analysis, writing – review & editing. Anett K. Leskó: investigation, resources. Haidar Hasan Mohammed: formal analysis, Writing – review & editing. Zoltán Németh: investigation, resources, formal analysis, supervision, writing – review & editing.

## Conflicts of interest

The authors declare no conflict of interest.

## Data Availability

The data supporting this article has been included within the article.
